# Synesthesia, at and near its borders

**DOI:** 10.3389/fpsyg.2013.00651

**Published:** 2013-09-26

**Authors:** Lawrence E. Marks, Catherine M. Mulvenna

**Affiliations:** ^1^John B. Pierce Laboratory, Department of Environmental Health Sciences, Yale School of Public Health, New Haven, CT, USA; ^2^Department of Psychology, Yale University, New Haven, CT, USA; ^3^Child Study Center, Yale University School of Medicine, New Haven, CT, USA

**Keywords:** synesthesia, cross-modal correspondence, cross-modal imagery, cross-modal memory, empathic perception, hallucination, Doppler illusion

## Introduction

In synesthesia, experiences in one domain evoke additional experiences in another, as when musical notes or letters of the alphabet evoke colors. Both the domains and their pairings are diverse. Indeed, Day's (2013) recently tabulated 60 types of synesthesia, each referring to a different combination of inducing and induced domains. The domains conjoined through synesthesia may belong to different sense modalities, as in music-color synesthesia, but may also belong to the same modality: In grapheme-color synesthesia, seeing printed letters or numbers evokes color experiences.

In music-color and grapheme-color synesthesia, the inducing stimuli are *perceptual*, reflecting culture-specific categories (notes of the Western musical scale, letters of the alphabet) learned by synesthetes and non-synesthetes alike. Synesthesia may be triggered not only by sounds, tastes, smells, and pains, but also by more complex signals: words(e.g., Simner, 2007), emotional states (e.g., Ward, 2004), and even personalities (e.g., Novich et al., 2011). Analogously, the domains of synesthetic responses too can range widely. The composer Rimsky-Korsakoff “saw” the key of D-major as golden (Myers, 1914), while a grapheme-personification synesthete reported, “Ts are generally crabbed, ungenerous creatures” (Calkins, 1893; p. 454). Other phenomena, however, such as cross-modally evoked images or memories, are not typically considered examples of synesthesia.

In this article, we briefly describe half a dozen illustrative cases that border on traditional forms of synesthesia: cross-modal correspondence, cross-modal imagery, sensory (cross-modal) autobiographical memory, empathic perception, hallucination, and the Doppler illusion. Do any or all of the six constitute forms of synesthesia? The answer depends, we suggest, on the framework for characterizing synesthesia. Consequently, after describing the six phenomena, we sketch three frameworks that differ in how they characterize these phenomena relative to prototypical forms of synesthesia. Several investigators, taking different perspectives and coming to different conclusions, have already considered possible relations to synesthesia in three of the six: cross-modal correspondence (Martino and Marks, 2001; Deroy and Spence, 2013); cross-modal imagery (Craver-Lemley and Reeves, 2013; Spence and Deroy, 2013); and empathic perception (Fitzgibbon et al., 2010; Rothen and Meier, 2013).

## Six at the borders: synesthesia's far and near kin?

### Cross-modal correspondence

Cross-modal correspondences pervade not only several forms of traditional synesthesia but also, importantly, the experiences of individuals typically deemed non-synesthetic (Marks, 1975, 1978; Spence, 2011). Even non-synesthetes perceive high-pitched vs. low-pitched sounds to resemble bright vs. dark colors—the resemblances evident in various tasks of cross-modal comparison (Marks, 1975; Ward et al., 2006). Where music-color synesthetes *see* brighter colors in high-pitched notes (e.g., “gold, yellow and white moving … like a rippling stream”: Mulvenna and Walsh, 2005; p. 399), people lacking the induced *qualia* of synesthesia nevertheless *recognize* cross-modal similarities.

Cross-modal correspondences often reflect alignments between bipolar dimensions, such as higher pitch being associated with greater lightness, greater brightness, higher vertical location, and smaller size (e.g., Karwoski et al., 1942; Wicker, 1968; Marks, 1974, 1989; Ward et al., 2006). Several auditory-visual correspondences reveal themselves in young children (Marks et al., 1987; Mondloch and Maurer, 2004) and infants (Lewkowicz and Turkewitz, 1980; Walker et al., 2010; Haryu and Kajikawa, 2012), as well as in denizens of disparate cultures: Members of a remote, semi-nomadic, preliterate desert-tribe in southern Africa, having virtually no contact with Western culture, nevertheless overwhelmingly matched lighter gray colors to higher-pitched tones—thereby revealing pitch-lightness correspondence (Mulvenna, 2012).

The tendency for non-synesthetes to perceive similarities between experiences in different domains, despite the absence of secondary *qualia*, has been called “synesthetic thinking” (Karwoski et al., 1942) and “weak synesthesia” (Martino and Marks, 2001), consistent with the notion that cross-modal correspondence reflects general perceptual and cognitive processes. Further, by capitalizing on cross-modal correspondences, synesthesia too presumably capitalizes on these general processes of perception and cognition (e.g., Karwoski et al., 1942; Marks, 1978; Ward et al., 2006).

### Cross-modal imagery

Compared to cross-modal correspondence, which can lack induced *qualia*, cross-modal imagery is nearer, phenomenologicaly, to prototypical synesthesia. In cross-modal imagery, as in synesthesia, stimulation in one modality may arouse mental images in another, although cross-modal imagery exhibits greater voluntary control (e.g., Karwoski and Odbert, 1938). Synesthetic responses commonly arise *automatically*, without requiring effort and being under relatively little control (e.g., Mattingley et al., 2001). By comparison, some non-synesthetes can voluntarily conjure up images, for example, imagining colors while listening to music (Karwoski et al., 1942). In some instances, there may be an especially intimate connection between visual imagery and prototypical sound-color synesthesia. Karwoski and Odbert (1938) inferred that a small subset of their subjects experienced visual imagery that could be modulated by music—a phenomenon that seems more automatic (less voluntary) than typical visual imagery, albeit less automatic (more voluntary) than synesthesia. Perhaps music-modulated imagery bears an especially close connection to traditional music-color synesthesia.

### Sensory autobiographical memory (proust phenomenon)

Marcel Proust (1922) famously described the floods of detailed, sensory memories from childhood evoked by tasting a tea-soaked *madeleine*. Sensory, autobiographical memory of this sort has been dubbed the “Proust phenomenon” in honor of the eponymous author. In the Proust phenomenon, odors or flavors in particular evoke strong sensory-based memories of associated events experienced in childhood (Chu and Downes, 2000). Proustian memory resembles traditional synesthesia, but also differs from it—resembling synesthesia in the automatic manner in which sensory experiences evoke memory images, but differing in the episodic character of the memories. In this regard, the sensory *qualia* of traditional synesthesia seem more “semantic” than “episodic.”

### Empathic perception: pain, touch, couvade syndrome

In several respects, empathetic perception strongly resembles synesthesia. In empathic pain, seeing or hearing evidence of another person's pain or discomfort produces analogous pain or discomfort (e.g., Jackson et al., 2005). Similarly, seeing another person being touched may produce an analogous tactile sensation—often called “mirror touch” (e.g., Banissy and Ward, 2007). Although the inducing stimuli come from another modality—typically, vision or hearing—the mechanisms underlying empathic perception presumably rely on an underlying within-domain equivalence: where implicitly-recognized sensations evoke sensory experiences of the same or similar kind, perhaps through merging constructs of “self” and “other.”

Possibly related to empathic pain is the *couvade syndrome*, which refers to a set of empathic symptoms (such as nausea, vomiting, and abdominal pain) reported by the partners of pregnant women. The couvade syndrome appears to be fairly common, having a reported prevalence of about 22% (Lipkin and Lamb, 1982), roughly five times that of traditional synesthesia (Simner et al., 2006).

A feature that distinguishes empathic perception from traditional forms of synesthesia is the very characteristic that makes empathy empathic—the intrinsic equivalence between the emotional qualities of the inducing and induced experiences. In traditional forms of synesthesia, however, as when sound evokes visual color or shape, the inducing and induced sensations not only reflect different domains but are also usually related more abstractly, even “metaphorically” (Rothen and Meier, 2013).

### Hallucination

A hallucination is a “percept-like experience which (a) occurs in the absence of appropriate stimulus, (b) has the full force or impact of the corresponding (real) perception, and (c) is not amenable to direct and voluntary control by the experiencer” (Slade and Bentall, 1988; p. 23). Several of these attributes also characterize synesthesia. Hallucinations may involve any of the senses (Ohayon, 2000) and are easily distinguished as “perceptions not confirmed by others” (Ohayon, 2000; p. 154). Some types of hallucinations, though not all, may fall near the borders of synesthesia. Thus, synesthetic experiences commonly include colors and shapes (Day, 2013). Analogously, “simple hallucinations,” often triggered by migraines or hallucinogens (Ermentrout and Cowan, 1979), commonly include simple recurring shapes and patterns, or ‘form constants (Klüver, 1966), which apparently reflect patterns of neural activation in visual cortex (Ermentrout and Cowan, 1979).

No longer considered explicitly pathological, hallucinations are now generally treated as independent perceptual phenomena (Romme and Escher, 1989; see Strauss, 1969). Auditory verbal hallucinations (hearing voices), for instance, occur in about 13% of adults in the general population (Beavan et al., 2011), and their presence does not correlate significantly with psychopathology (Johns et al., 2002; Sommer et al., 2010). Although the strongest predictor of psychopathology in hallucinations is distress over their content or possible basis (Romme and Escher, 1989; Chadwick and Birchwood, 1994; Beavan et al., 2011), distress is rarely associated with synesthetic experience.

### The doppler illusion

Day's (2013) table listing 60 types of synesthesia includes only one type that is explicitly intra-modal, namely, grapheme-color synesthesia. We note here another phenomenon in which sensory experiences in one domain induce experiences in the same modality, the “Doppler illusion,” reported by Neuhoff and McBeath (1996): When a tone increases continuously in intensity over time (as though a sound-emitting source were approaching at constant velocity) but maintains a constant sound frequency, observers nevertheless report hearing the tone's pitch to increase as loudness increases. Neuhoff and McBeath dubbed the illusory increase in pitch the “Doppler illusion” because a sound source approaching at constant velocity will produce, at the observer's location, an elevated, albeit constant, sound frequency (the physical Doppler effect). But might we not also call the Doppler illusion a case of intra-modal (loudness-pitch) synesthesia?

## Synesthesia: continuous, discrete, pluralistic?

If, as the term implies, synesthesia is first and foremost a “conjoining of experiences,” then one might construe several or perhaps all six of our cases as examples of synesthesia. If, on the other hand, synesthesia is defined more narrowly, for example, by requiring it to include *qualia* and to arise automatically and consistently, then fewer cases would make the cut. Recently, the first author outlined three theoretical frameworks—monism, dualism, and pluralism—that, in different ways, characterize how synesthesia could relate to borderline perceptual and conceptual phenomena like the six just described (Marks, 2011, 2012).

Synesthetic *monism* refers to the notion that synesthesia may appropriately be considered a spectrum or continuum. Using this framework, traditional forms of synesthesia, such as music-color and grapheme-color, serve as prototypes, residing at the high end of the continuum, with weaker forms, such as cross-modal correspondence, residing toward the low end. In the present examples, music-modulated imagery, hallucinations, empathic perceptions, Proustian evoked memories, and the Doppler illusion might lie at various loci between the ends of the synesthetic spectrum—although the differences amongst them suggest that the hypothesized spectrum is multidimensional.

The other two frameworks both distinguish sharply between synesthesia and non-synesthetic forms of perception and conception. *Dualism* posits a simple dichotomy between the two categories, with synesthesia incorporating both traditional forms (e.g., sound-color, grapheme-color, word-flavor, taste-shape) and others (e.g., grapheme-personification, ordinal sequence-spatial sequence), and non-synesthesia incorporating cross-modal correspondence, cross-modal imagery, and hallucination. How to categorize other borderline examples, such as empathic perception, Proustian memory, and the Doppler illusion, however, is less clear.

Like dualism, the third framework, *pluralism*, explicitly distinguishes synesthetic from non-synesthetic experiences, but rests on the additional assumption that synesthesia is well characterized as (appropriating James's, 1890; p. 224, expression) “a teeming multiplicity.” Like monism, synesthetic pluralism recognizes that some forms of synesthesia, such as music-color, are better exemplars than are others, such as empathic perception and the Doppler illusion. In the pluralistic view, however, the broad category of synesthesia itself contains a cornucopia of distinct sub-categories, lacking common denominators but perhaps linked one to another along the lines suggested by Wittgenstein's (1953) notion of family resemblance.

Critical, in our view, to choosing amongst frameworks is characterizing the role of phenomenal experience in defining synesthesia; many investigators judge this role to be significant (see, e.g., the exchange among Cohen Kadosh and Terhune, 2012; Eagleman, 2012; Simner, 2012a, b). Monism in particular relies substantially on the notion that phenomenal experience plays a central, and ineluctable, role in characterizing synesthesia. Alternatively, jettisoning phenomenology may be conducive to pluralistic frameworks that rely on mechanism-based distinctions amongst multiple forms of synesthesia. And jettisoning phenomenology may be especially conducive to dualistic frameworks that rely on a mechanism-based distinction between synesthesia and borderline phenomena—perhaps akin to distinguishing mechanistically between rhinovirus-induced sniffles and pollen-induced seasonal nasal allergies.

But we ask, Are phenomenal experiences (*qualia*) analogous to sneezes?
